# Dysferlinopathy in Switzerland: clinical phenotypes and potential founder effects

**DOI:** 10.1186/s12883-015-0449-3

**Published:** 2015-10-06

**Authors:** Jens A. Petersen, Thierry Kuntzer, Dirk Fischer, Maja von der Hagen, Angela Huebner, Veronika Kana, Johannes A. Lobrinus, Wolfram Kress, Elisabeth J. Rushing, Michael Sinnreich, Hans H. Jung

**Affiliations:** Department of Neurology, University Hospital Zürich, Frauenklinikstrasse 26, 8091 Zürich, Switzerland; Nerve-Muscle Unit, Neurology Service, Department of Clinical Neurosciences, Lausanne University Hospital (CHUV), Lausanne, Switzerland; Department of Neurology, University Hospital Basel, Basel, Switzerland; Abteilung Neuropäriatrie, Dresden, Germany; Klinik für Kinder- und Jugendmedizin, Medizinische Fakultät Carl Gustav Carus, Technische Universität Dresden, Dresden, Germany; Institute of Neuropathology, University Hospital Zürich, Zürich, Switzerland; Department of Pathology, University Hospital Geneva, Geneva, Switzerland; Institure of Human Genetics, University of Würzburg, Würzburg, Germany

## Abstract

**Background:**

Dysferlin is reduced in patients with limb girdle muscular dystrophy type 2B, Miyoshi myopathy, distal anterior compartment myopathy, and in certain Ethnic clusters.

**Methods:**

We evaluated clinical and genetic patient data from three different Swiss Neuromuscular Centers.

**Results:**

Thirteen patients from 6 non-related families were included. Age of onset was 18.8 ± 4.3 years. In all patients, diallelic disease-causing mutations were identified in the *DYSF* gene. Nine patients from 3 non-related families from Central Switzerland carried the identical homozygous mutation, c.3031 + 2T>C. A possible founder effect was confirmed by haplotype analysis. Three patients from two different families carried the heterozygous mutation, c.1064_1065delAA. Two novel mutations were identified (c.2869C>T (p.Gln957Stop), c.5928G>A (p.Trp1976Stop)).

**Conclusions:**

Our study confirms the phenotypic heterogeneity associated with *DYSF* mutations. Two mutations (c.3031 + 2T>C, c.1064_1065delAA) appear common in Switzerland. Haplotype analysis performed on one case (c. 3031 + 2T>C) suggested a possible founder effect.

## Background

Dysferlin (DYSF) is a transmembrane protein linked to sarcolemmal repair mechanisms. Autosomal recessive mutations in the *DYSF* gene cause muscular dystrophies (MD): a limb girdle MD, the so-called LGMD 2B, with onset in the proximal lower limbs [1], and two distal MD, one initially affecting the gastrocnemius muscle during early adulthood, Miyoshi myopathy (MM) [2], and the other involving muscles of the anterior compartment (DMAT) [3]. Disease severity ranges from asymptomatic mutation carriers with elevated serum CK levels [4] and exercise intolerance [5] to severe functional disability [6]. Muscle weakness with pelvic girdle involvement on clinical examination distinguishes LGMD from MM. Other common phenotypes include a “proximo-distal” phenotype, characterized by simultaneous distal and proximal weakness onset [6]. Of note, a recent MRI study suggests that all patients have radiographic evidence of proximo-distal muscle involvement, regardless of the clinical phenotype [7].

The *DYSF* gene maps to chromosome 2p12-p14, contains 55 exons and is transcribed as an 8.5 Kb major transcript mainly expressed in skeletal and cardiac muscles. The protein product is a 230 kDa molecule involved in muscle fibre repair [8, 9]. More than 400 *DYSF* mutations have been described in the Leiden muscular dystrophy database (http://www.dmd.nl); however, a mutational hot spot has not been detected [10–12]. Ethnic clusters have been described in Jews from Libya [13] and the Caucasus region [14], as well as in the Italian [15] and Spanish [16] populations. Four mutations account for 60 % of all mutations in Japanese patients with MM [17]. Interestingly, the type of mutation does not correlate with phenotypic features. In fact, the same mutation has been found to be associated with a broad spectrum of inter—and intra-familial clinical phenotypes [18–22].

Within this range of clinical and genetic heterogeneity, we sought to determine frequently encountered *DYSF* gene mutations and phenotypes in the Swiss population and to uncover possible founder effects.

## Methods

### Subjects

The medical records of patients of Swiss origin with progressive muscle weakness, and *DYSF* mutations, who were admitted to Neuromuscular Centres in Basel, Lausanne and Zurich between 1989 and 2015 were anonymized and reviewed. All patients provided their written informed consent for genetic analysis. Clinical data collected included age and symptoms at onset, disease duration, family pedigrees, and muscle strength according to the Medical Research Council (MRC) scale. In addition, CK levels, muscle biopsy and mutational analysis results were evaluated. Electromyography, electrocardiography, echocardiography, pulmonary function and muscle MRI data were reviewed when available. The study was performed in accordance with the Declaration of Helsinki and approved by the local ethics committee (Kantonale Ethikkommission Zürich, KEK-ZH-Nr. 2015–0036).

### Genetic analysis

Sequence analysis was performed using bidirectional fluorescent sequencing of all 55 exons of the *DYSF* gene, either in the Service de Cardiologie, Hôpital Cochin CHU Paris, France, or at the Institute of Human Genetics, University of Würzburg, Germany (Reference sequence: NM_003494.3). Multiplex ligation-dependent probe amplification (MLPA) was used in one case.

To evaluate the hypothesis that the *DYSF* mutation c.3031 + 2T>C is due to a founder effect, we performed haplotype analysis, as described previously, with 6 polymorphic microsatellite markers on chromosome 2p13.2 flanking the *DYSF* gene (*D2S327*, *D2S2113*, *D2S292*, *D2S291*, *D21S2110* and *D2S2111*) [23].

## Results

### Clinical data

Thirteen patients were included in the study. Of those, 9 patients were from three non-related families from Central Switzerland (Canton of Schwyz). The clinical data of 10 patients from 6 different families are summarized in Tables 1 and 2. Three patients from central Switzerland (C-6, D-10, D-11) provided blood for haplotype analysis but refused clinical evaluation. Consanguinity was not reported and the parents of all patients were healthy. Mean disease onset was in late adolescence or early adulthood (18.7 ± 4.5 years). Early manifestations (Table 1) included leg pain, steppage gait, Gowers’ sign and difficulty running and jumping. Mean CK levels were 12500 ± 14400 IU/L (range 2200–46350, normal value for our laboratory < 167 U/l, Table 1).

**Table 1:** Clinical data of Swiss dysferlinopathy patients

Family-patient	Origin (Canton)	Sex/age (years)	Age at onset (years)	Symptoms at onset	CK level (IU/L)	Cardiac ultrasound/ECG	Pulmonary function	EMG
A-1	Fribourg	M/58	23	Steppage gait	>2.300	normal/normal	FVC 55 %	n.d.
A-2		F/54	28	Difficulty walking on tiptoes	>2.300	normal/normal	normal	Tibialis anterior muscle: myopathic, fibrillations
B-3	Aargau	F/33	15	Difficulty running	3.800	normal/normal	normal	Biceps brachii muscle: myopathic
C-4	Schwyz	M/27	17	Difficulty skiing	>23.000	normal/normal	normal	Vastus lateralis muscle:
				(downhill) and running;				myopathic, fibrillations,
				leg pain				positive sharp waves
C-5		F/24	17	Difficulty walking on	>15.000	normal/normal	normal	Tibialis anterior muscle:
				tiptoes; leg pain				myopathic
C-6		F/31		*blood sample provided*				
				*for haplotype analysis;*				
				*consultation rejected*				
C-7		M/16	14	Difficulty toe walking	46.350	normal/normal	normal	Vastus lateralis muscle:
								myopathic; positive
								sharp waves
C-8		F/54	16	Leg weakness	n.d.	n.d.	n.d.	Extensor carpi radialis
								muscle: myopathic
D-9	Schwyz	F/45	16	Gowers’ sign	>2.200	normal/normal	n.d.	Vastus lateralis muscle:
								myopathic; fibrillations,
								positive sharp waves
D-10		F/46		*blood sample provided*				
				*for haplotype*				
D-11		M/48		*analysis;*				
				*consultation rejected*				
E-12	Thurgau	M/28	20	Difficulty jumping,	>10.600	normal/normal	normal	n.d. (syncope during
				Leg pain				needle insertion)
F-13	Schwyz	M/22	21	Leg weakness	>9.000	normal/normal	normal	Gastrocnemius muscle:
								myopathic; fibrillations, positive sharp waves

*M* male, *F* female, *FVC* Forced vital capacity, *n.d.* not done, normal CK < 167 U/l. “myopathic” refers to small, short, polyphasic potentials

**Table 2:** Phenotype of Swiss dysferlinopathy patients

Family-patient	A-1		A-2		B-3	C-4		C-5		C-7		C-8	D-9	E-12		F-13
Age at onset	23		28		15	17		17		14		16	16	20		21
Age at examination/years after onset	46/23	55/32	45/17	52/24	30/15	20/3	27/11	18 /1	26/9	15/1	18/4	44/28	36/20	27/7	31/11	21/0
Facial muscles	5/5	5/5	nd	nd	5/5	5/5	5/5	5/5	5/5	5/5	5/5	5/5	5/5	5/5	5/5	5/5
Head extension	5/5	5/5	5/5	5/5	nd	5/5	5/5	5/5	5/5	5/5	5/5	4/4	nd	5/5	nd	5/5
Shoulder elevation	5/5	5/5	5/5	5/5	5/5	5/5	5/5	5/5	5/5	5/5	5/5	nd	nd	nd	nd	5/5
External arm rotation	3/3	0/0	4+/4+	5-/5-	nd	5/5	nd	nd	nd	5/5	nd	0/0	nd	nd	nd	nd
Internal arm rotation	3/3	nd	nd	nd	nd	5/5	nd	nd	nd	5/5	nd	nd	nd	nd	nd	nd
Arm adduction	3/3	0/0	nd	nd	3/3	5/5	nd	nd	nd	5/5	4/4	nd	nd	5/5	4+/4+	nd
Arm abduction	1/1	0/0	4+/4+	5/5	3/3	5/5	5-/5-	5/5	5/5	5/5	4/5	0/0	4/4	5/5	4-/4-	5/5
Arm flexion	nd	0/0	4+/4+	4/4	4/4	5/5	5-/5-	5/5	5/5	5/5	4/4	0/0	4/4	5/5	4-/4-	5/5
Arm extension	1/1	1/1	4+/4+	4+/4+	3/3	5/5	5-/5-	5/5	5/5	5/5	4+/4+	0/0	4/4	5/5	4/4	5/5
Wrist supination	4+/4	nd	nd	nd	nd	nd	nd	nd	5/5	5/5	nd	nd	nd	nd	nd	nd
Wrist pronation	4+/3	3/3	4+/4+	5/5	nd	nd	nd	nd	5/5	5/5	nd	nd	nd	nd	nd	nd
Hand flexion	nd	3+/3+	4+/4+	5/5	4/4	5/5	5-/5-	5/5	5/5	5/5	4/4	4+/4	5-/5-	5/5	4+/4+	5/5
Finger flexion	3/3	3-/3-	4+/4+	4/4	4/4	5/5	5-/5-	5/5	5/5	5/5	5/5	4+/4	5-/5-	5/5	4+/4+	5/5
Hand extension	5/5	4+/4	nd	nd	4/4	5/5	5-/5-	5/5	4/4	5/5	4/4	4+/4	5-/5-	5/5	4+/4+	5/5
Finger extension	5/5	4-/4-	5-/5-	5/5	4/4	5/5	5-/5-	5/5	4/4	5/5	4/4	4+/4	5-/5-	5/5	4+/4+	5/5
Thumb abduction	nd	4/4	4+/4+	4+/4+	nd	5/5	nd	nd	nd	5/5	nd	nd	nd	nd	nd	nd
Thumb extension	5/5	nd	nd	nd	nd	5/5	nd	nd	nd	5/5	nd	nd	nd	nd	nd	nd
Hip adduction	2/2	0/0	3/3	2+/2+	4/4	5/5	nd	5-/5-	nd	5/5	2/2	nd	4/4	nd	4+/4+	nd
Hip extension	5/5	4+/4+	4+/4+	4-/4-	nd	4/4	4-/4-	5-/5-	nd	5/5	4/4	1/1	4/4	nd	4-/4-	5/5
Knee extension	2/2	0/0	2+/2	2/2	2/2	5/5	4/4	5/5	5-/5-	5/5	4/4	3/3	3/3	5/5	4/4	5/5
Knee flexion	2/2	0/0	3+/3+	3-/3-	2/2	5/5	4-/4-	5/5	3+/3+	5/5	4/4	4/4	4/4	5/5	4-/4-	5/5
Foot extension	0/0	0/0	2/2	2/2	3/3	5/5	2/2	5/5	2/2	4/4	4/4	0/0	5-/5-	4/4	4/4	4/4
Toe extension	0/0	0/0	3-/3-	nd	nd	nd	nd	nd	nd	nd	nd	1/1	nd	3/3	nd	nd
Foot flexion	5/5	3/3	1/1	1/1	1/1	4-/4	2/1	5-/5-	3/3	4/4	3/3	1/1	4+/4+	4/4	4/4	3/4
Toe flexion	4+/4+	3/3	1/1	1/1	nd	nd	nd	nd	nd	nd	nd	4/4	nd	4-/4-	nd	nd

Muscle strength grades according to Medical Research Council (MRC) scale (right/left); nd: not done

The clinical phenotype could be determined in 10 patients (Table 2). In all patients, the facial muscles were spared. Four of the patients from Central Switzerland (C-4, C-5, C-7, F-13) presented without arm weakness a few years after the onset of symptoms (Table 2). All patients had distal lower limb flexor weakness and 2 (C-7, F-13) also showed distal lower limb extensor weakness. Three (C-4, C-5, C-7) patients had proximal lower limb weakness and therefore were classified as the “proximo-distal” (PD) phenotype [6]. Another patient (F-13) demonstrated isolated distal lower limb weakness (foot flexion > extension) and was classified as the MM phenotype. Three patients (C-4, C-5 and C-7) were followed for 11, 9 and 4 years, respectively (Table 2). All developed distal upper limb extensor weakness with 2 (DC-4, C-7) additionally showing distal upper limb flexor and proximal upper limb weakness. Two patients from central Switzerland (C-8, D-9) were examined 20 and 28 years after onset and demonstrated proximal and distal upper and lower limb flexor and extensor weakness (Table 2). Patient D-9 experienced a relatively “benign” course with muscle strength grade of at least 3 on the MRC scale (Table 2). One patient from the Canton Thurgau (E-12) presented 7 years after onset with proximal and distal lower limb flexor and extensor weakness, eventually developing distal flexor and extensor and proximal upper limb weakness (Table 2). A patient from the Canton Aargau (B-3) presented with severe distal lower limb flexor, proximal lower limb flexor and extensor, distal upper limb flexor and extensor and proximal upper limb weakness 15 years after onset. Two patients from the Canton Fribourg were initially examined 23 (A-1) and 17 (A-2) years after onset, respectively. Patient A-1 had generalized weakness, pronounced in the distal lower limb extensors, and over the course of 9 years developed proximal lower and upper limb palsies. Patient A-2 had generalized weakness, more pronounced in the distal lower limb flexors, that progressive minimally over 7 years. Interestingly, members of family C presented with a proximo-distal phenotype early in the course and members of family C and patient F-13 were notable for the lack of upper limb weakness during the early phases of the disease. Later in the course, all patients manifested a typical LGMD phenotype; however, the distribution of weakness was heterogeneous (Table 2). The DMAT phenotype was not observed in our cohort.

The use of mobility aids showed the following timeline: 2 patients required canes for walking assistance (A-1, A-2) at the age of 35 and 49 years, respectively (disease duration until first use of canes 12 and 21 years, respectively), and both were eventually wheelchair-bound (17 and 24 years after disease onset; 5 and 3 years after first cane use). Patient B-3, at age 29, 14 years after disease onset, used a wheelchair intermittently (B-3), and patient C-8 was wheelchair-bound, at age 43, 27 years after disease onset.

### Cardiac, pulmonary, MRI and EMG data

Electrocardiography and ECG were normal in all 9 patients who underwent examination, the longest disease duration being 35 years (Table 1). Pulmonary function was normal in 6 out of 7 patients, with restrictive lung disease documented in patient A-1 (Table 1; initial pulmonary function testing performed after 29 years of disease duration). In 3 patients, muscle MRI showed fatty replacement of the lower limb muscles. In all three patients (A-2, E-12 and F-13), the short head of the biceps femoris and the sartorius and the gracilis muscles were symmetrically spared or appeared less affected than other muscles. In patients E-12 and F-13, the semitendinosus, the tibialis anterior and the extensor digitorum longus muscles also seemed less affected, while edema was suspected in selected lower limb muscles. In another patient (C-7), the pelvic and the lower limb muscles appeared normal. EMG was myopathic with short and small potentials in all 8 patients examined, with spontaneous activity found in 5 cases (Table 1).

### Pathological features and protein studies

A total of 5 surgical muscle biopsies were available (Table 3) for evaluation. In 2 patients, biopsies were not available because they were taken in 1986 and 1977. In 6 patients, the genetic diagnosis was made without prior biopsy. The mean age at biopsy was 28.25 ± 13.7 years, while the mean disease duration at biopsy was 8.3 ± 9.8 years. Samples were removed from the trapezius, quadriceps and gracilis muscles. All patients exhibited dystrophic changes with an increased spectrum of myofiber diameter and variable fibrosis. In one case with a LGMD phenotype (B-3), striking inflammatory changes were observed within the endomysium. Western blots (WB) and/or immunohistochemistry (IHC) were performed in 6 samples (WB only in 2 samples, IHC only in 3 samples, WB and IHC in a single sample). On WB, dysferlin was totally absent in all samples tested, whereas by IHC, dysferlin was judged absent in 2 cases and reduced in two.

**Table 3:** Histological and biochemical data of Swiss dysferlinopathy patients

Family-patient	Disease duration at biopsy (y)/muscle/findings	WB Dysf/immunohistochemistry
A-1	26/trapezius/atrophic fibers; slight fibrosis; fatty infiltration	absent/nd
A-2	*Biopsy in 1986; histopathological data na*	absent/nd
B-3	1/vastus lateralis/atrophic fibers; fibre size variability;/re- and degenerating fibers; necrotic fibers; endo- and perimysial fibrosis; inflammation	absent/reduced
C-4	3/vastus lateralis/de- and regenerating fibers,	nd/absent
	whorled fibers, myophagocytosis, endomysial fibrosis	
C-5 to C-7	*Genetic testing without prior biopsy*	
C-8	*Biopsy in 1977; na*	
D-9	20/vastus lateralis/focal fiber atrophy, hypertrophy,	nd/reduced
	necrotic fibers, increased internalized nuclei	
D-10, D-11	*Genetic testing without prior biopsy*	
E-12	1/gracilis/re- and degenerating fibers,	nd/absent
	myophagocytosis, fibrosis, COX-neg. fibers	
F-13	*Genetic testing without prior biopsy*	

*Nd* not done, *WB Dysf* result of the presence or absence of dysferlin by Western-blot analysis

### Genetic studies

Genetic data are summarized in Table 4. In 13 patients, 2 disease-causing mutations on separate alleles were identified. Nine patients from 3 non-related families from Central Switzerland (Canton Schwyz) carried the identical splice site mutation, c.3031 + 2T>C, 7 were homozygous (Table 4). Three patients from 2 different families from the Cantons of Fribourg and Aargau carried a heterozygous frameshift mutation c.1064_1065delAA, and 2 members from family A, harbored the heterozygous nonsense mutation c.2217C>A (Table 4).

**Table 4:** Dysferlin mutations in Swiss patients

Family-patient	Exon/Intron	DNA	Effect of mutation
A-1	ex12	c.1064_1065delAA	p.Lys355ArgfsX4
	ex23	c.2217C>A	p.Tyr739X
A-2	ex12	c.1064_1065delAA	p.Lys355ArgfsX4
	ex23	c.2217C>A	p.Tyr739X
B-3	ex12	c.1064/1065delAA	p.Lys355ArgfsX4
	ex25-27		Deletion exon 25-27
C-4	IVS28	c. 3031 + 2T>C	Abn. Spli.
	IVS28	c. 3031 + 2T>C	Abn. Spli.
C-5	IVS28	c. 3031 + 2T>C	Abn. Spli.
	IVS28	c. 3031 + 2T>C	Abn. Spli.
C-6	IVS28	c. 3031 + 2T>C	Abn. Spli.
	ex27	c. 2869C>T	p.Gln957X
C-7	IVS28	c. 3031 + 2T>C	Abn. Spli.
	IVS28	c. 3031 + 2T>C	Abn. Spli.
C-8	IVS28	c.3031 + 2T>C	Abn. Spli
	IVS28	c.3031 + 2T>C	Abn. Spli
D-9	IVS28	c. 3031 + 2T>C	Abn. Spli.
	IVS28	c. 3031 + 2T>C	Abn. Spli.
D-10	IVS28	c. 3031 + 2T>C	Abn. Spli.
	IVS28	c. 3031 + 2T>C	Abn. Spli.
D-11	IVS28	c. 3031 + 2T>C	Abn. Spli.
	IVS28	c. 3031 + 2T>C	Abn. Spli.
E-13	Ex53	c.5928G>A	p.Trp1976Stop
	Ex53	c.5928G>A	p.Trp1976Stop
F-13	IVS28	c. 3031 + 2T>C	Abn. Spli.
	IVS28	c. 3031 + 2T>C	Abn. Spli.

Novel mutations appear in bold; *Ex* exon, *IVS* intervening sequence

Two novel mutations were identified in this patient set. Patient C-6, who was compound heterozygous for the c.3031 + 2T >C mutation on one allele, showed a novel mutation, c.2869C>T on the other allele. Patient E-12 carried the novel homozygous c.5928G > A mutation.

**Fig. 1:**
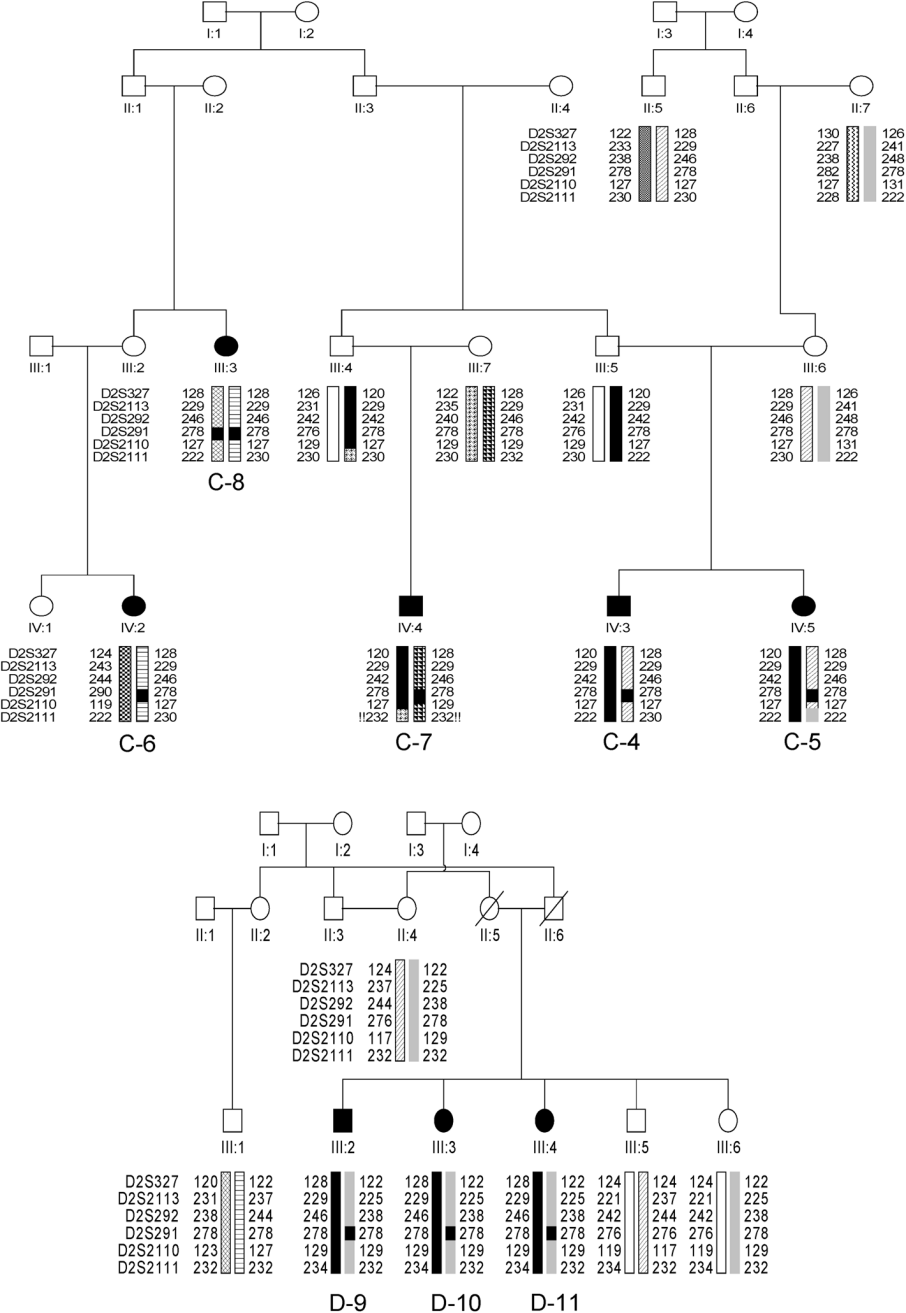
Haplotype Analysis. Haplotype analysis using six microsatellite markers flanking the *DYSF* gene in families C and D*.* The *DYSF* gene is located between markers *D2S292* and *D2S291*. In all patients carrying the homozygous c.3031 + 2T>C mutation, the adjacent marker *D2S291* also shows a homozygous genotype (allele 178) suggesting that this mutation may be a founder mutation in these two families. Accordingly, in patient C-6 carrying a compound heterozygous *DYSF* mutation (c.3031 + 2T>C and c.2869C>T), the marker D2S291 shows a heterozygous genotype (278–290)

Haplotype analyses using markers flanking the *DYSF* gene revealed homozygosity for at least one nearest marker (*D2S291*) in all patients from the two apparently unrelated C and D families carrying the homozygous c.3031 + 2T>C mutation, suggesting a possible founder mutation (See Fig. 1). In patient C-7, carrying a compound heterozygous *DYSF* mutation (c.3031 + 2 T >C and c.2869C>T), marker *D2S291* shows a heterozygous genotype (Fig. 1).

## Discussion

Evaluation of the present cohort of 6 families brings new genetic information about dysferlinopathy in Switzerland, and novel data regarding a potential founder effect in Central Switzerland. The fact that unrelated families with identical mutations come from the same region indicates that these mutations are common, and indeed, haplotype analysis of 2 families carrying the mutation c.3031 + 2T>C suggests a possible founder effect for this mutation in Switzerland. The limitations of this study are twofold: the analysis is limited by the small cohort size and 3 out of 13 patients had not been examined clinically.

From a clinical point of view, the onset age in early adulthood confirms previous findings [6, 7, 24]. In addition, late-onset [25] or congenital forms were not encountered [26]. Three out of 4 patients from Central Switzerland presented with the PD phenotype, while another patient with the same mutation was considered to represent the MM phenotype. Upper limb weakness was not a presenting feature in the current cohort. Some reports suggest that the proximo-distal phenotype occurs in up to one third of dysferlinopathy cases [6]. Of note, 9 out of 10 patients developed distal upper limb weakness later in the disease course, which has been frequently reported [27–29]. Muscle MRI is known to reveal fatty replacement of distal and proximal lower limb muscles in the early stages of dysferlinopathy, including patients with clinically isolated proximal or distal involvement [7, 30]. However, an adolescent patient with a MM phenotype (C-7) showed a normal pelvic and whole lower limb muscle MRI, similarly to a patient described with isolated hyperCKemia [31].

Interestingly, we found clinical phenotype variability within families and within unrelated carriers with similar mutations. Such variability has already been reported in Libyan Jews [13] and was found to be particularly relevant in a 6-generation, highly consanguineous family originating from an isolated village in Dagestan [32]. This observation may indicate that to date, unknown genetic or environmental modifiers play a role in influencing the clinical phenotypes of the mutated *DYSF* gene [22]. Accordingly, it would be interesting to study micro-RNAs known to be involved in many biological processes, including epigenetic changes. Other cardinal features of our cohort were very high CK levels, as previously described [7], the absence of cardiac impairment (nevertheless encountered in few patients [6, 25, 33, 34] and present in a knock out mouse model [35]), and the risk of developing respiratory failure late in the disease course [36, 37], as observed in one of our patients.

EMG showed myopathic changes and pathologic spontaneous activity. In one case, the biopsy revealed striking endomysial inflammation, in line with frequently observed inflammatory changes in dysferlinopathy and other muscular dystrophies [18, 38]. Immunosuppressive treatment, however, fails to improve muscle weakness [39]. It has been postulated that dysferlin may be linked to membrane repair and/or inflammatory activation [40], and in a few cases, splice site mutations disrupting dysferlin are known to produce inflammation [18]. Conversely, most of our patients with splice mutations showed no evidence of inflammation on muscle biopsy, indicating the lack of clear correlation between inflammation and genotype. On Western blot (WB) 3 cases showed a consistent reduction in dysferlin, as has been previously described [41].

In dysferlinopathies, genotype is not predictive of disease severity, and genotype-phenotype correlations are not clear-cut [15, 42]. However, correlation has been found between certain geographical areas and the age of onset, notably in Japanese and Italian MM patients [17], who develop symptoms earlier than patients with LGMD 2B [10]. The most severely affected patients in our study (Patients A-1 and A-2, described previously [12]) carried the truncating mutation c.2217C>A (p.Tyr739X) with a disease duration of 17 and 24 years, respectively, before the patients became wheelchair-bound. One of the older patients (C-8), carrying the c.3031 + 2T>C homozygous mutation, became wheelchair bound after a disease duration of 27 years, and patient D-9 showed relatively mild impairment 20 years after onset. However, in all other patients carrying this mutation, disease duration was shorter; thus precluding longitudinal comparisons. A longer follow-up is necessary to assess the onset and time-course of the various disabling symptoms.

## Conclusion

Our study confirms the broad phenotypic heterogeneity, often accompanied by markedly elevated CK levels, associated with *DYSF* mutations in the Swiss population. Due to a potential founder effect in the past, two mutations appear to be endemic in central Switzerlans. These findings are important for genetic counselling and should facilitate targeted molecular diagnosis of dysferlin deficiency in patients of Swiss origin.
